# A primer for choosing, designing and evaluating registered reports for qualitative methods

**DOI:** 10.12688/openreseurope.15532.1

**Published:** 2023-01-31

**Authors:** Veli-Matti Karhulahti, Peter Branney, Miia Siutila, Moin Syed

**Affiliations:** 1 Faculty of Humanities and Social Sciences, University of Jyväskylä, Jyväskylä, Finland; 2 Department of Psychology, University of Bradford, Bradford, UK; 3 Faculty of Humanities, University of Turku, Turku, Finland; 4 Department of Psychology, University of Minnesota, Twin Cities, USA

**Keywords:** guidelines, open science, transparency, qualitative research, registered reports

## Abstract

Registered reports are a publication format that involves peer reviewing studies both before and after carrying out research procedures. Although registered reports were originally developed to combat challenges in quantitative and confirmatory study designs, today registered reports are also available for qualitative and exploratory work. This article provides a brief primer that aims to help researchers in choosing, designing, and evaluating registered reports, which are driven by qualitative methods.

## Introduction

Registered reports (“RRs”, Chambers & Tzavella, 2022) are a modification of the scientific publication process that aims to shift publication decisions from being based on the nature of the results of the study and towards rigorous conceptualization and design. In so doing, RRs improve transparency and the timeliness of peer review. The FORTT (2022) glossary defines RRs as a two-stage process, where peer review occurs at study design (Stage 1) and, if ‘in-principle-accepted’ (IPA), study completion (Stage 2). While RRs were originally developed for research following the hypothetico-deductive method ( Chambers, 2013), today they are also open to explorative and qualitative studies ( Branney *et al*., 2022). In fact, some qualitative RRs are already emerging (e.g., Stage 1, Topor *et al*., 2022) and at least two have already been published (Stage 2: Karhulahti *et al*., 2022; Xiao, 2022).

**Figure 1.**
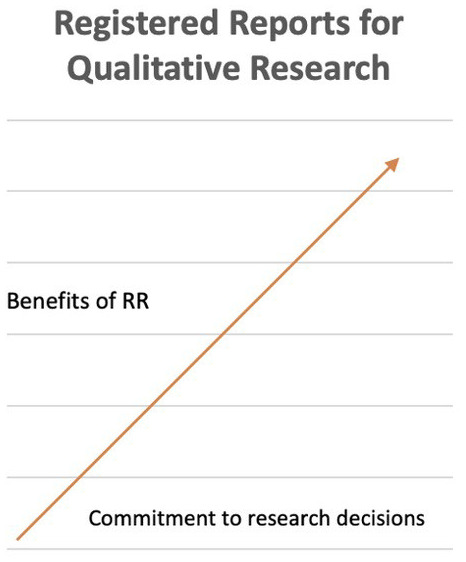
The benefits of registered reports (RRs) correlate with the possibilities to commit to decisions.

As RRs are likely to be unfamiliar to those using qualitative methods, this is a brief primer on choosing, designing and evaluating RRs for qualitative research. This primer is principally for people who design and conduct research using qualitative methods, although it may also be useful for editors and undergraduate and graduate students. As a rule of thumb, we propose the following: *the more qualitative researchers can commit to research decisions before the study, the more benefits they will harvest by using the RR format* ( Figure 1). In other words, not all qualitative designs benefit from using the RR format equally, but some may benefit significantly.

## Choosing RRs for qualitative research

### Frontloading peer review

Choosing to carry out a research project in the RR format allows one to receive peer review feedback in the design phase. This can be helpful especially for early career researchers and teams with limited qualitative expertise. Feedback alone can be a good reason for choosing the RR format if the study timeline allows. Projects that are carried out under severe time pressure should carefully consider whether they wish to use the RR format, as carefully reviewing the study design usually takes time (see Evans *et al*., 2023). On the other hand, RRs submitted via *Peer Community in Registered Reports* (PCI RR) can schedule their review to save time, or even start generating data before completing Stage 1 by lowering the reported control level ( PCI RR, 2022).

### Negotiation…

*…with fellow researchers and stakeholders*

In frontloading the design, an RR gives legitimacy to researchers and stakeholders spending time transparently negotiating the study design, including uncertainties and delayed decision making. This could mean that the design is collaborative and each person involved has agency in decision making. For example, in Button *et al*’s (2020) model for consortium based empirical undergraduate dissertations, the academics collaboratively write the study protocol and main hypothesis; once the new academic year commences, the dissertation students join the consortium and they collaboratively develop the protocol further, with each student having responsibility for adding one secondary hypothesis. While qualitative studies are unlikely to have testable hypotheses, this grassroots training model can be used in qualitative RRs to negotiate better research questions and the study design in ways that give those involved agency.

*…with Journal or review platform*

The RR format allows negotiating research decisions with editors before carrying out the study. As journals and review platforms have many explicit and implicit policies regarding the studies they publish, using the RR format allows pursuing an agreement with them at Stage 1. This can save valuable time and resources compared to traditional publication processes, which sometimes produce month/year-long (desk) rejection loops.

### Transparency

Due to the more flexible nature of decision-making in qualitative (vs. quantitative) research, authors may need to apply for a permission to make changes between Stage 1 and 2. Although this is generally against the philosophy of hypothesis-testing RRs and increases editorial labor, it is also a robust means for documenting the expected and unexpected events in the research process.

## Designing qualitative RRs

### Analysis

Methods of analysis should be written in such detail that editors and reviewers understand what will be done (Stage 1) and what was done (Stage 2). Researchers should also clearly explain how they plan to report the data and results. In qualitative studies, methods tend to include flexible elements (see Haven & Van Grootel, 2019). Such elements should be identified and planned, i.e. if researchers cannot decide between multiple alternative analyses, rules of decision making can be stated instead (e.g., if X then we Y). If biases are of epistemological concern, it is also possible for teams to apply masked analyses ( Dutilh *et al*., 2021).

### Data access & stewardship

Qualitative research can include a wide range of different data types such as audio, images, videos, and written text. All data types are suitable for RRs. Qualitative data are likely to present ‘legitimate sensitivities’ to participants' privacy ( Branney *et al*., 2019), even through the ‘innocent collection of details’ ( Branney *et al.*, 2017). Therefore, it is important to outline and negotiate data sharing plans at an early stage, and RRs facilitate this process ( Karhulahti, 2022a). Two current trends contribute to the importance of these efforts.

The first trend is the digitization of our research and data protection legislation, which means that data can have a short life ( Fang *et al*., 2013) and will be impossible to access without clear consent and data sharing agreements between all organizations (compared to reading through letters or documents in a filing cabinet, for example where researchers had only a single copy of their data but still shared it, see Craig & Reese, 1973). Organizations, for example, may be unable to grant access to archeologists of the future. The second trend is the range of data sharing and open data policies of research bodies, such as funders and professional bodies (see Riley *et al*., 2019). From 2013, the UK Economic and Social Research Council had a policy that data should be available for reuse within three months of grant completion ( ESRC, 2013). In turn, the British Psychological Society has a ‘as open as possible; as closed as necessary’ position statement on open data ( BPS, 2020). 

It is less common for qualitative studies to share data openly, especially in human research. On the other hand, data sharing is also possible for qualitative studies, and should be encouraged whenever possible (for an overview, see DuBois *et al*., 2018). The Qualitative Data Repository was developed for precisely this purpose, but authors can also consult national and international repositories for assistance in sharing procedures. Anonymization (or pseudonymization), consent procedures, and controlling data reuse are preferably carried out in collaboration with archives and experts ( Karhulahti, 2022b). Clear reasons should be provided if data are non-shareable. Notably, some qualitative data is not collected but rather generated (e.g., interviews), in which case the researchers’ own involvement should be recognized, for instance, via positionality statements.

### Ethics approval and IRBs

Different countries and universities apply different processes for reviewing research ethics, and each Stage 1 submission should communicate how they meet the standards of their country or institution. When needed, ethics approval for RRs should generally be applied before Stage 1 submission, but parallel and post-IPA approvals are negotiable as well in case in inflexible committees (see Figure 3 in Chambers & Tzavella, 2022; also, PCI RR, 2022). Authors can discuss with journal (or PCI RR) representatives when applying for approval, being explicit to each about the amendments recommended by each process. If an ethics approval sets critical limits to a planned design, authors can also consult RR representatives before submission and report those limits in their Stage 1 proposal to minimize misunderstandings in peer review.

### Hypotheses

It is uncommon (but not impossible) for qualitative research designs to test hypotheses. On the other hand, it is also possible to set hypotheses without testing them. These “qualitative hypotheses” (QHs) can be used in a similar way as positionality statements, i.e. to report the team’s prior beliefs and hypothetical biases, which can affect the study design and its procedures. So far, at least two RRs ( Karhulahti *et al*., 2022; Topor *et al*., 2022) have used qualitative hypotheses. Unlike positionality statements, QHs are based on previous data, literature, and theory, which have influenced the study design and may influence data interpretation, thus being akin to ‘priors’ in Bayesian statistical designs (see Andrews & Baguely, 2017).

### Positionality

Because qualitative research is often highly interpretive and reflective, it is important to disclose the position(s) from which the interpretations and reflections are made. It may be useful to have separate positionality statements for data generation (e.g., interviews) and analysis (e.g., coding). The APA Journal Article Reporting Standards for qualitative research, for example, recommend describing the researchers, how their perspectives were used in methodological integrity of the data collection and analysis, and their understanding of the conclusions ( APA, 2020; Levitt *et al*., 2018).

### Research questions

As qualitative research is usually nonconfirmatory, one of the most important parts of study design is the formation of useful research questions. Specific types of qualitative data and analyses are often suitable for producing answers only to specific types of research questions, for which authors should carefully assess these relationships in their design. In general, good research questions ensure that the produced answers will contribute to the field.

### Sample (size)

There are no universal sampling rules for qualitative studies, but samples should always be justified. Different justifications apply to different types of data and methods. When justifying the nature and size of a sample, authors might also apply e.g., saturation, where the analytic process defines the sample during data generation (e.g., Low, 2019). In such cases, however, it can still be useful to preregister estimations, which facilitate editorial work and increase transparency.

## Evaluating qualitative RRs

### Evaluating cost-benefit (Stage 1)

Because successful RRs receive in-principle acceptance after Stage 1, one of the special features in evaluating them is to assess whether the study is worth carrying out. Importantly, this is not the same as “impact”, but rather the degree to which a study can contribute, given resources. The value of expected findings needs to be assessed against the current knowledge, e.g. do the findings have potential to produce useful contributions to knowledge or improve the understanding of a phenomenon? In most cases, this means carefully evaluating the research questions and their match with the generated data as well as applied analyses. Because many authors are pre-committed to asking specific research questions (e.g., for funding bodies), even small contributions can be worth pursuing. Primarily, reviewers should help authors to maximize the contribution potential of their resources. 

### Evaluating interpretations (Stage 2)

As authors must clearly spell out how they will report the data and results at Stage 1, reviewers should start by assessing whether the plan was followed and if not, are possible deviations justified. A key difference between qualitative and other RRs is the former’s strong affiliation with interpretative and reflective methodology. This means that reviewers have less control over the production of results compared to e.g., statistically driven studies. Because interpretations may not always be fully reproducible (recall positionality), reviewers balance their assessment between ensuring that authors communicate their interpretations clearly, back up the interpretations with data, and draw reasonable conclusions from the analysis (see Josselson, 2004).

### Evaluating language (Stage 1 & 2)

Qualitative studies, which are typically exploratory by nature, should rarely use confirmatory language. Likewise, as qualitative studies less commonly reach high generalizability, language should be used with care when discussing the findings in general contexts. Reviewers should pay special attention to the language of discussion and conclusions at Stage 2.

### Evaluating data/materials (Stage 1 & 2)

Peer reviewers should generally be provided secure access to data at Stage 2. Materials used or produced in analysis should be included as an appendix to the shared data whenever possible; for instance, coding manuals and documentation for establishing reliability/trust should always be shared (when applicable). On the other hand, data sharing is not a binary question, but usually parts of the data can be shared whereas other parts (e.g., with potential personal identifiers) cannot ( Syed, 2022). These dimensions must be assessed case by case. Agreeing upon a clear plan for data and material sharing at Stage 1 and assessing them at Stage 2 are an important part of the evaluation process.

## Use cases and discussion

Registered reports are becoming increasingly popular across fields, and many of their benefits also apply to qualitative methods. So far, our own experiences as authors and evaluators ( Topor *et al*., 2022; Xiao, 2022) serve as practical use cases, which have demonstrated the potential of registered reports for qualitative methods. As authors ( Karhulahti *et al*., 2022), we witnessed a significant improvement of our research design at Stage 1 due valuable review feedback, and the registered report format did not conflict with the flexibility characteristic to qualitative research, but we were also allowed to make non-registered choices, such as reporting themes through a case format that turned out optimal after analysis. As evaluators ( Karhulahti *et al*., 2022; Topor *et al*., 2022; Xiao, 2022), we witnessed exceptional motivation to support the authors because feedback at Stage 1 can have a fundamental positive effect on the study design and the quality of the research—this cannot happen to the same degree in publication formats, which are reviewed *after* the study has already been carried out. In sum, based on our experience, registered reports allow authors and evaluators to “play in the same team,” of which the present primer is also a practical example: authors and their evaluators having teamed up for follow-up collaboration.

We hope this brief primer is helpful for authors, editors, and reviewers involved in qualitative registered reports, and we look forward to updating these recommendations along with our accumulating experience and knowledge.

## Ethics and consent

Ethical approval and consent were not required.

## Data Availability

No data are associated with this article.
