# Supplementary material for: A primer for choosing, designing and evaluating registered reports for qualitative methods
Source: Open Res Eur. 2023 Jul 10;3:22. Originally published 2023 Jan 31. [Version 2] doi: 10.12688/openreseurope.15532.2 (PMC10445887; doi:10.12688/openreseurope.15532.2)
Supplement: Peer review history

# Peer review: Reviewer response for version 2

**DOI:** 10.21956/openreseurope.17581.r33612

**Published:** 2023-07-25

**Authors:** Corina Logan

**Affiliations:** 1 Max Planck Institute for Evolutionary Anthropology, Leipzig, Germany

It looks great! I'm really excited to have this resource available to continue to make registered reports more useful for qualitative researchers.

Are sufficient details provided to allow replication of the method development and its use by others?

Yes

Is the description of the method technically sound?

Yes

Are the conclusions about the method and its performance adequately supported by the findings presented in the article?

Yes

Is the rationale for developing the new method (or application) clearly explained

Yes

If any results are presented, are all the source data underlying the results available to ensure full reproducibility?

No source data required

Reviewer Expertise:

Animal behavior, open research practices, registered reports

I confirm that I have read this submission and believe that I have an appropriate level of expertise to confirm that it is of an acceptable scientific standard.

# Peer review: Reviewer response for version 2

**DOI:** 10.21956/openreseurope.17581.r33613

**Published:** 2023-07-11

**Authors:** Bogdana Huma

**Affiliations:** 1 Department of Language, Literature, and Communication, Faculty of Humanities, Vrije Universiteit Amsterdam, Amsterdam, The Netherlands

I would like to thank the authors for the rigour with which they have undertaken the revisions, leading to an improvement of the paper.

Are sufficient details provided to allow replication of the method development and its use by others?

Yes

Is the description of the method technically sound?

Yes

Are the conclusions about the method and its performance adequately supported by the findings presented in the article?

Yes

Is the rationale for developing the new method (or application) clearly explained

Yes

If any results are presented, are all the source data underlying the results available to ensure full reproducibility?

No source data required

Reviewer Expertise:

Open Science in qualitative research; qualitative methodologies; conversation analysis

I confirm that I have read this submission and believe that I have an appropriate level of expertise to confirm that it is of an acceptable scientific standard.

# Peer review: Reviewer response for version 1

**DOI:** 10.21956/openreseurope.16790.r30776

**Published:** 2023-03-13

**Authors:** Bogdana Huma

**Affiliations:** 1 Department of Language, Literature, and Communication, Faculty of Humanities, Vrije Universiteit Amsterdam, Amsterdam, The Netherlands

This article provides a brief overview of how registered reports can be adapted for and adopted in qualitative research. In the context of the expansion of the Open Science movement, this is a very timely and important discussion. I enjoyed reading this article and hope that my comments below will help to strengthen the arguments put forward therein.

 Comments

- First, I wonder about the relevance of Figure 1? The content of the figure is nicely summarised in paragraph 2 as “the more qualitative researchers can commit to research decisions before the study, the more benefits they will harvest by using the RR format”. This makes the figure somewhat redundant. Also, the figure suggests a simple direct linear relationship between the benefits of using RR and commitments to prior decisions, which might be a little misleading, unless there is evidence that can back up this particular type of relationship.
- In the first paragraph of the section “Frontloading peer review” the authors state that “Choosing to carry out a research project in the RR format allows one to receive peer review feedback in the design phase. This can be helpful especially for early career researchers and teams with limited qualitative expertise”. It would be useful to provide some backup for this last claim, in the form of, for example, citations, concrete examples etc. If no evidence for this exists yet, then perhaps the claim should be reformulated as a hypothetical statement.
- The section on “Negotiation…with fellow researchers” features a nice example of how RR allow multiple parties to participate in negotiating the design of a QUANTITATIVE study. Given that the audience for this article may be interested mainly in how this applies to QUALITATIVE research, an example of how negotiating works in a qualitative study would be welcome.
- In the section on “Data access & stewardship”, the claim is made that “It is less common for qualitative studies to share data openly, especially in human research”. This is certainly true, but it would be beneficial to include some context for why this is the case. Also, it might be worth highlighting that some qualitative approaches, such as conversation analysis, include data sharing practices embedded in their methodologies. In fact, in conversation analysis, data have always been shared in many different ways and that is a defining characteristic of this approach – for details, see Joyce et al. (2022).
- I found the section on ‘Use cases and discussion’ particularly valuable because if briefly presented concrete examples of RR. I was wondering whether the authors could elaborate this section, and, in particular, give concrete examples of how the feedback received at Stage 1 has helped improve the articles.
- Lastly, I have a general observation about the use of methodological terms in this paper. Often methodological discussions (including those about Open Science) are written up in terms that apply mainly to quantitative research methods and that is problematic because those terms mobilise some assumptions about research that do not apply to all qualitative methodologies. Therefore, it may be relevant for the authors to go through the paper and consider some of the vocabulary choices made and their alternatives. I can see that this has already been considered on page 4 when the issue of data “collection” vs “generation” is mentioned. Other terms that may benefit from reflection are: “sample”, “results” (vs analysis or findings) etc.

Are sufficient details provided to allow replication of the method development and its use by others?

Yes

Is the description of the method technically sound?

Yes

Are the conclusions about the method and its performance adequately supported by the findings presented in the article?

Yes

Is the rationale for developing the new method (or application) clearly explained

Yes

If any results are presented, are all the source data underlying the results available to ensure full reproducibility?

No source data required

Reviewer Expertise:

Open Science in qualitative research; qualitative methodologies; conversation analysis

I confirm that I have read this submission and believe that I have an appropriate level of expertise to confirm that it is of an acceptable scientific standard.

## References

[1] : Should we share qualitative data? Epistemological and practical insights from conversation analysis. *International Journal of Social Research Methodology* .2022; 10.1080/13645579.2022.2087851 1-15 10.1080/13645579.2022.2087851

# Response

**Published:** 2023-06-30

**Authors:** Veli-Matti Karhulahti

We are very thankful for the feedback and detailed suggestions for improvement. Because we applied various solutions to different points, they are listed below one by one.  

 1. We have removed Figure 1, which we agree did not add new information.

 2. We have removed the sentence, which did not have empirical support yet.

 3. We have clarified how frontloading example applies to qualitative work too.

 4. We have highlighted differing traditions across qualitative areas with reference to Joyce et al. (2022) and added context, within the limits of keeping the sections at similar length.

 5. We have clarified the uses cases section and expanded it.

 6. We have additionally edited “sample” and “results” sentences, which we agree benefit from terminological diversification. In future revisions of this article, it might be worth writing a completely new section about terminological differences (but we felt this should be left for future more comprehensive versions that will systematically expand each section after we have more experience of completed qualitative RRs).  

 One more thank you and all the best wishes, On behalf of all authors, Veli-Matti Karhulahti

# Peer review: Reviewer response for version 1

**DOI:** 10.21956/openreseurope.16790.r30777

**Published:** 2023-02-02

**Authors:** Corina Logan

**Affiliations:** 1 Max Planck Institute for Evolutionary Anthropology, Leipzig, Germany

This is a timely "How to" article for researchers conducting qualitative research in how to write, evaluate, update, and use registered reports to their advantage. Registered reports are new for qualitative research and the experiences of these authors who have been involved in the process as authors and evaluators is particularly useful. I am sure this will primer will be welcomed widely.

 I have only a few minor comments:

 Transparency > change "apply for a permission" to "apply for permission"

 Transparency > "Although this is generally against the philosophy of hypothesis-testing RRs". It is my understanding that authors with Stage 1 IPA at various journals can contact their editor after IPA to discuss changes they might need to make, even for RRs that involve hypothesis testing.

 Ethics approval > change "ethics approval for RRs should generally be applied before Stage 1 submission" to "ethics approval for RRs should generally be obtained before Stage 1 submission"

 Ethics approval > change "but parallel and post-IPA approvals are negotiable as well in case in inflexible committees" to "but parallel and post-IPA approvals are negotiable as well in case the committee is inflexible"

 Hypotheses > what a great idea to liken QHs to priors!

 Evaluating cost-benefit (stage 1) > I love the distinction between usefulness and impact. Change "study can contribute, given resources" to "study can contribute, given the available resources"

 Evaluating interpretations (Stage 2) > "are possible deviations justified". Perhaps also add whether these deviations were pre-approved by the editor, as you state above.

 Use cases and discussion > "Stage 1 due valuable review feedback" to "Stage 1 due to valuable review feedback"

 Use cases and discussion > change "same degree in publication formats" to "same degree in other publication formats"

 All my best,

 Corina Logan

Are sufficient details provided to allow replication of the method development and its use by others?

Yes

Is the description of the method technically sound?

Yes

Are the conclusions about the method and its performance adequately supported by the findings presented in the article?

Yes

Is the rationale for developing the new method (or application) clearly explained

Yes

If any results are presented, are all the source data underlying the results available to ensure full reproducibility?

No source data required

Reviewer Expertise:

Animal behavior, open research practices, registered reports

I confirm that I have read this submission and believe that I have an appropriate level of expertise to confirm that it is of an acceptable scientific standard.

# Response

**Published:** 2023-06-30

**Authors:** Veli-Matti Karhulahti

We are very thankful for the feedback and detailed suggestions for improvement.  We have made all the suggested changes, without exceptions, and fully agree that these have improved the article. 

 One more thank you and all the best wishes, On behalf of all authors,

 Veli-Matti Karhulahti
